# CT imaging in children with non-severe asthma and cough-variant asthma: functional spasm or structural remodeling?

**DOI:** 10.3389/fmed.2026.1755012

**Published:** 2026-03-16

**Authors:** Li Wang, Chunying Liu, Yinghong Fan, Wanmin Xia, Cheng Xie, Yanru Liu, Pinli Zou, Qianqian Li, Tao Ai

**Affiliations:** 1 Department of Pediatric Respiratory Medicine, Chengdu Women’s and Children’s Central Hospital, School of Medicine, University of Electronic Science and Technology of China, Chengdu, China; 2 Department of Radiology, Chengdu Women’s and Children’s Central Hospital, School of Medicine, University of Electronic Science and Technology of China, Chengdu, China

**Keywords:** airway remodeling, children, cough-variant asthma (CVA), CT, non-severe asthma

## Abstract

**Objective:**

To investigate the role of CT-measured bronchial wall thickness and area in detecting airway remodeling in children with non-severe asthma and cough-variant asthma.

**Methods:**

A retrospective review was conducted on 140 children who were assigned to the non-severe asthma (AS, *n* = 51), cough-variant asthma (CVA, *n* = 50) and control (CTL, *n* = 39) groups at Chengdu Women’s and Children’s Central Hospital from January 2023 to June 2025. Clinical characteristics, pulmonary function, and CT-measured parameters were compared across the groups.

**Results:**

The wall area (WA), wall thickness (WT), wall area percentage (WA%), and wall thickness percentage (WT%) were measured in the third and fourth segments of right upper lobe apical segmental bronchus (RB1), right lower lobe posterior basal segmental bronchus (RB10) and left lower lobe posterior basal segmental bronchus (LB10). These parameters were significantly higher in both the AS and CVA groups compared to the CTL group (*p* < 0.05). No statistically significant differences in age, gender, white blood cell count (WBC) or eosinophil count (EOS) were observed between the patient group (AS and CVA) and CTL group. Based on pulmonary function results, children with asthma or CVA were divided into groups with or without small airway dysfunction (SAD) and with or without obstructive ventilatory disorder (OVD). The comparative analysis showed that the majority of CT-measured parameters did not exhibit any statistically significant differences when comparing the SAD group to the non-SAD group, or the OVD group to the non-OVD group.

**Conclusion:**

In conclusion, the CT results suggest that there may be airway remodeling in non-severe asthma and CVA patients in the early stages of the disease. Although patients with CVA differ from those with classic asthma in their clinical presentation and pulmonary function, they may share the same pattern of airway remodeling.

## 1 Introduction

Asthma, a prevalent chronic respiratory disorder in the pediatric population, continues to exhibit a rising global incidence, particularly in developing nations. This trend poses a growing burden on health and quality of life of children (1). The principal pathological features of asthma include airway inflammation, airway hyperresponsiveness, and airway remodeling. Airway remodeling involves structural alterations in the airway wall, which encompass epithelial injury, basement membrane thickening, smooth muscle hyperplasia, and subepithelial fibrosis. These changes can lead to irreversible airway narrowing and progressive decline in lung function (2). In the pediatric population, airway remodeling may initiate earlier in life and exert long-term consequences on disease outcomes. Therefore, the early identification and assessment of remodeling are critical for clinical management.

The evaluation of airway remodeling has traditionally depended on invasive methods, notably bronchial biopsy, which is significantly limited by ethical and safety considerations. High-resolution computed tomography (HRCT), as a non-invasive imaging technique, is widely used to assess structural airway changes in asthma by quantifying metrics such as bronchial wall thickness (WT), bronchial dilation, and the bronchial wall area percentage (WA%). These parameters reflect the extent of airway remodeling. For instance, increased WT is often associated with basement membrane thickening and airway inflammation and is particularly prominent in cases of severe and therapy-resistant childhood asthma (3, 4). Multiple studies have demonstrated that high-resolution CT (HRCT) can detect bronchial wall thickening in children with severe asthma. This structural alteration cannot be solely attributed to bronchospasm and may instead represent underlying airway inflammation or remodeling (4). A quantitative CT analysis study in children with severe asthma demonstrated that patients exhibited significantly greater WT, WA%, and air trapping index compared to healthy controls. These structural abnormalities were inversely correlated with lung function parameters, including forced expiratory volume in one second (FEV_1_) and forced vital capacity (FVC) (5). Another study reported that in pediatric asthma patients who did not respond to omalizumab therapy, three-dimensional bronchial wall analysis revealed increased WT associated with airway remodeling, which may contribute to the observed treatment resistance (6). Furthermore, recent studies utilizing machine learning to HRCT analysis have corroborated that metrics such as WT and bronchiectasis can accurately distinguish children with severe asthma from healthy controls and assess disease severity (7).

While these findings underscore the potential of CT imaging in revealing airway remodeling in severe and therapy-resistant asthma, existing research has been predominantly confined to these populations. Comprehensive analyses are notably lacking for non-severe asthma and cough-variant asthma (CVA), which constitute a substantial proportion of pediatric asthma cases. To address this gap, this study aims to investigate the diagnostic value of CT metrics for identifying airway remodeling in children with non-severe asthma and cough-variant asthma, to provide an imaging rationale for early clinical intervention.

## 2 Materials and methods

### 2.1 Study population

**FIGURE 1:**
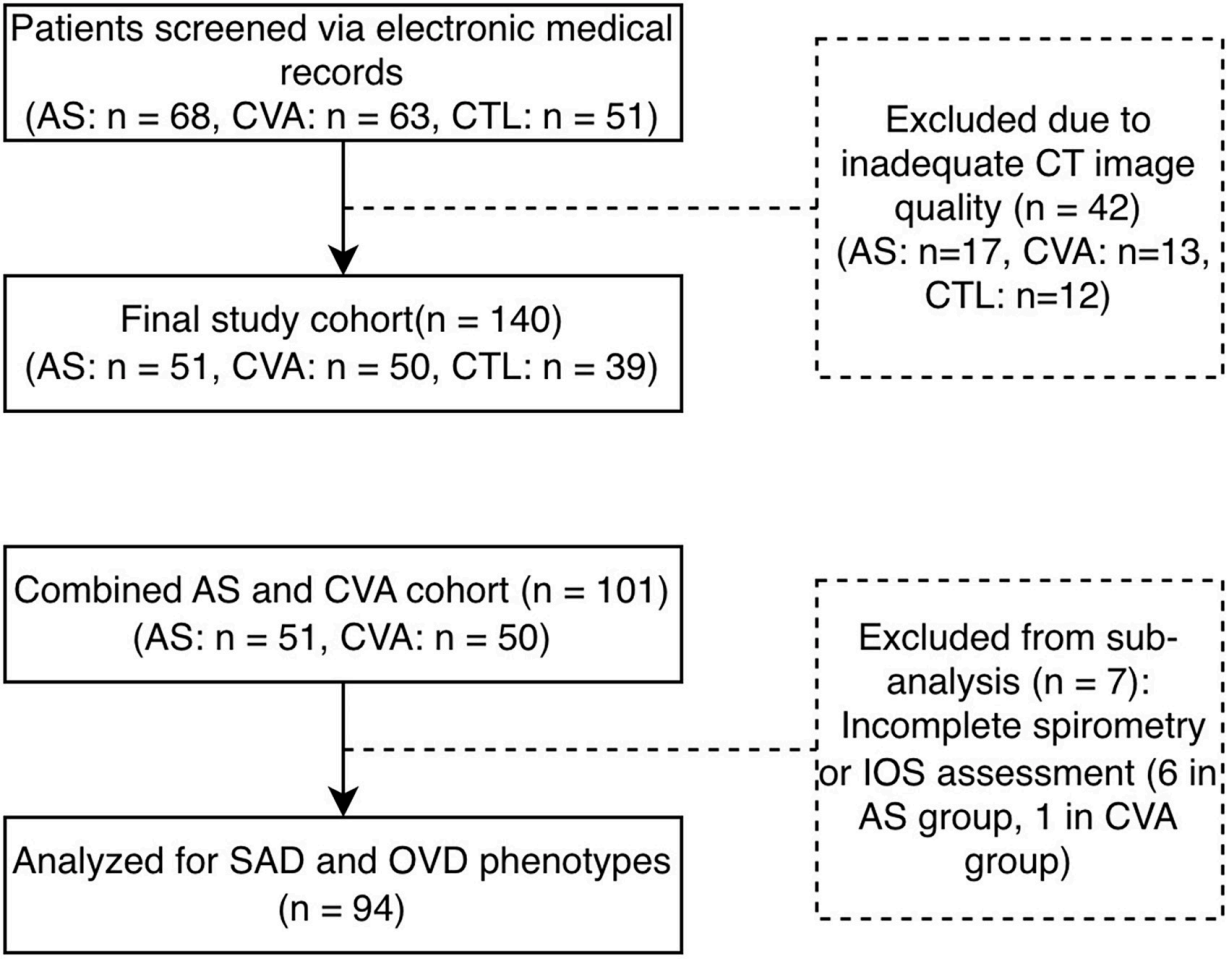
Flowchart of participant enrollment, exclusion, and phenotypic sub-grouping. AS, non-severe asthma; CVA, cough variant asthma; CTL, control; SAD, small airway dysfunction; OVD, obstructive ventilatory disorder; IOS, impulse oscillometry.

A total of 182 children were initially screened through a retrospective review of electronic medical records at Chengdu Women’s and Children’s Central Hospital between January 2023 and June 2025. After a rigorous quality control (QC) of chest CT images, 42 children were excluded due to motion artifacts (involving >1/3 of the target bronchial segments). Consequently, a final cohort of 140 children was enrolled in this study, comprising a non-severe asthma group (AS, *n* = 51), a CVA group (*n* = 50), and a control group (CTL, *n* = 39). The screening and enrollment process is detailed in Figure 1. Diagnoses for the AS and CVA groups were strictly based on the Chinese “Standardized Management of Pediatric Bronchial Asthma (2020 edition)” (8). The CTL group consisted of children presenting with chest pain who underwent chest CT and blood tests for clinical differential diagnosis (to rule out pneumothorax, pulmonary anomaly, or myocarditis) but were confirmed to have no respiratory or systemic pathologies. The exclusion criteria for AS and CVA groups were as follows: refractory asthma (9), congenital heart disease, immunodeficiency disorders, concurrent pneumonia or other pulmonary pathologies. All children were aged 4–16 years old. The studies involving human participants were reviewed and approved by the ethics board of Chengdu Women and Children Center Hospital (Ethical number: [2021]203). Written informed consent for the CT examinations was obtained from legal guardians as part of routine clinical care. For the purpose of this retrospective analysis, the requirement for additional study-specific informed consent was waived by the ethics committee.

### 2.2 High-resolution computed tomography (HRCT)

Computed tomography imaging was performed using a SIMENS 64-slice spiral CT scanner. The children were placed in the supine position, and the scan was acquired at full inspiration breath-hold to minimize variability due to respiratory motion. The scanning parameters were as follows: tube voltage, 80–100 kV; tube current, 80–130 mA; window width, 1500 HU; window level, −500 HU; matrix, 512 × 512; and slice thickness, 0.625 mm. All chest CT scans were performed at the end of a deep inspiration to ensure optimal lung inflation. To ensure image quality in young children (especially those aged 4–6 years), a standardized respiratory coaching protocol was implemented. Prior to the scan, a 5–10 min training session was conducted with each child and their legal guardian to practice breath-holding maneuvers. For the CT acquisition, the automated voice prompts of the scanner were disabled to allow for personalized timing. Instead, the radiologic technologist provided manual voice commands based on real-time observation of the child’s breathing pattern. To minimize anxiety and improve cooperation, parents were permitted to remain in the room with appropriate radiation protection.

### 2.3 Quantitative CT metrics of the bronchi

Given the retrospective design, CT image quality varied across patients, and localized artifacts occasionally affected visualization of individual bronchi. To minimize measurement bias while preserving data representativeness, image quality control was performed at the airway-segment level rather than excluding entire scans solely due to focal artifacts. All CT datasets were independently reviewed by two experienced radiologists blinded to clinical information. During manual airway measurements, bronchial segments affected by motion artifacts, image noise, partial volume effects, or unclear wall boundaries were considered non-measurable and recorded as missing values. To ensure analytical reliability, a predefined exclusion threshold was applied at the patient level. Subjects with more than one-third of target bronchial measurements deemed non-measurable were excluded from the final analysis. Disagreements between reviewers were resolved by consensus. This predefined quality control strategy was implemented to balance measurement validity with avoidance of unnecessary case exclusion.

The target bronchi for parameter quantification included the 3rd and 4th order bronchi in the following segments: the apical segment of the right upper lobe (RB1), the posterior basal segments of the right lower lobe (RB10) and the left lower lobe (LB10). The directly measured metrics included the inner diameter (ID) and outer diameter (OD) of the bronchi. The bronchial wall thickness (WT), wall thickness percentage (WT%), wall area (WA), and wall area percentage (WA%) were calculated using the formulas in BOX 1. During data acquisition, the short-axis view of the target bronchus was obtained, ensuring the image was as circular as possible. For each bronchus, the longitudinal and transverse diameters corresponding to both the ID and OD were measured. The respective ID and OD values were then calculated by averaging these two perpendicular measurements. The specific calculation method is as follows:

BOX 1 **Formulas for Bronchial Wall Measurement Parameters**

$W\,T=\frac{O\,D-I\,D}{2}$

$W\,T\%=\frac{W\,T}{O\,D}\times 100\%$

$W\,A=\frac{\pi \times O\,D^{2}}{4}-\frac{\pi \times I\,D^{2}}{4}$

$W\,A\%=\frac{4\,W\,A}{\pi \times O\,D^{2}}\times 100\%$

### 2.4 Pulmonary function test

Spirometry or impulse oscillometry (IOS) was performed for AS group and CVA group within the 24-h period preceding or following the chest CT examination. The equipment and operational protocol were consistent with those described in our previous study (10). Spirometry parameters were collected as follows: the FEV_1_, FVC, FEV1/FVC, forced expiratory flow rates at 25%, 50%, and 75% of FVC (FEF_25_, FEF_50_, FEF_75_), and maximum mid-expiratory flow (MMEF) were obtained from spirometry. The total respiratory impedance (Z_5_), airway resistance at 5 Hz (R_5_) and 20 Hz (R_20_), the resistance difference (R_5_–R_20_), resonant frequency (Fres), and reactance at 5 Hz (X_5_) were collected from IOS.

### 2.5 Other indicators

Peripheral blood parameters, including white blood cell count (WBC), eosinophil count (EOS), eosinophil percentage (EOS%), and fractional exhaled nitric oxide (FeNO) levels, were obtained from all study participants (both patient groups and the healthy controls). The complete blood count analysis was performed on the same day as the chest CT examination. FeNO measurement was conducted within a 24-h window surrounding the CT scan (either before or after), strictly adhering to the operational standards outlined in the 2011 American Thoracic Society guidelines (11).

### 2.6 Statistical analysis

Data analysis was performed by SPSS 26.0 statistical software. Normally distributed continuous data are presented as mean ± standard deviation (x ± s), while non-normally distributed data are presented as median with interquartile range [M(Q1, Q3)]. For comparisons between two groups, the independent samples *t*-test was used for parametric data, and the Mann-Whitney U test was used for non-parametric data. Categorical data are expressed as frequencies (%) or counts (*n*=) and were compared using the Chi-square test.

## 3 Results

### 3.1 Comparison of clinical characteristics and CT parameters among groups

A total of 51 children with AS (33 males/18 females), 50 with CVA (28 males/22 females), and 39 healthy controls (25 males/14 females) were enrolled. The age range was 4–14 years for the AS/CVA groups and 4–16 years for the CTL group. Comparisons among the AS group, the CVA group, and the CTL group are summarized in Tables 1, 2.

**TABLE 1:** Comparison of clinical data and CT parameters among the AS group, CVA group, and control group.

Variables	AS *n* = 51	t/χ^2^	*P* ^¢^	CVA *n* = 50	t/χ^2^	*P* ^§^	CTL *n* = 39
Age	6.33 ± 2.37	−1.197	0.235	6.22 ± 2.41	−1.421	0.158	6.87 ± 1.72
Sex (M/F)	33/18	0.004	0.953	28/22	0.597	0.440	25/14
WBC	8.39 ± 3.19	0.512	0.61	8.59 ± 3.49	−1.421	0.158	7.87 ± 4.06
EOS%	4.41 ± 5.37	2.932	0.005	3.05 ± 2.55	−0.669	<0.001	0.02 ± 0.02
EOS	0.35 ± 0.51	1.593	0.117	0.24 ± 0.21	−4.263	0.056	0.12 ± 0.16
**Third-order bronchi of RB1**							
WA	12.76 ± 3.54	3.255	0.002	13.21 ± 3.40	−1.953	<0.001	10.15 ± 4.06
WT	1.03 ± 0.16	4.677	<0.001	1.09 ± 0.15	−3.850	<0.001	0.82 ± 0.26
WT%	0.42 ± 0.06	3.951	<0.001	0.44 ± 0.08	−6.111	<0.001	0.35 ± 0.09
WA%	0.66 ± 0.07	4.315	<0.001	0.68 ± 0.09	−4.797	<0.001	0.57 ± 0.12
**Fourth-order bronchi of RB1**							
WA	8.49 ± 2.89	3.360	0.001	8.54 ± 2.25	−4.825	<0.001	6.68 ± 1.96
WT	0.92 ± 0.20	3.531	0.001	0.94 ± 0.16	−4.020	<0.001	0.76 ± 0.19
WT%	0.48 ± 0.08	2.826	0.006	0.49 ± 0.08	−4.895	<0.001	0.43 ± 0.11
WA%	0.73 ± 0.08	2.820	0.006	0.73 ± 0.09	−2.831	0.006	0.66 ± 0.14
**Third-order bronchi of RB10**							
WA	13.22 ± 3.45	3.824	<0.001	12.61 ± 3.24	−2.714	0.008	10.57 ± 3.46
WT	1.02 ± 0.20	2.998	0.004	1.01 ± 0.20	−2.777	0.007	0.84 ± 0.24
WT%	0.40 ± 0.07	2.755	0.007	0.40 ± 0.08	−3.478	0.001	0.35 ± 0.10
WA%	0.63 ± 0.10	2.806	0.006	0.64 ± 0.09	−2.625	0.010	0.57 ± 0.14
**Fourth-order bronchi of RB10**							
WA	9.62 ± 3.21	2.998	0.004	9.82 ± 3.33	−2.649	0.010	10.57 ± 3.46
WT	0.96 ± 0.23	3.533	0.001	0.95 ± 0.20	−3.090	0.003	0.84 ± 0.24
WT%	0.46 ± 0.09	2.709	0.008	0.45 ± 0.08	−3.340	0.001	0.41 ± 0.11
WA%	0.70 ± 0.1	2.827	0.006	0.69 ± 0.09	−1.855	0.068	0.64 ± 0.12
**Third-order bronchi of LB10**							
WA	13.66 ± 3.66	3.745	<0.001	13.32 ± 3.52	−2.015	0.048	10.72 ± 3.35
WT	1.01 ± 0.17	3.720	<0.001	1.03 ± 0.19	−3.308	0.001	0.85 ± 0.21
WT%	0.38 ± 0.06	1.763	0.082	0.41 ± 0.08	−4.071	<0.001	0.35 ± 0.09
WA%	0.62 ± 0.08	1.908	0.060	0.64 ± 0.09	−2.571	0.012	0.57 ± 0.12
**Fourth-order bronchi of LB10**							
WA	9.60 ± 3.14	3.471	0.001	9.56 ± 2.59	−2.579	0.012	7.35 ± 2.46
WT	0.91 ± 2.46	2.682	0.009	0.96 ± 0.16	−3.634	0.001	0.79 ± 0.20
WT%	0.44 ± 0.09	0.375	0.709	0.45 ± 0.09	−3.959	<0.001	0.42 ± 0.11
WA%	0.68 ± 0.11	0.509	0.612	0.69 ± 0.12	−0.914	0.364	0.66 ± 0.13

RB1, right upper lobe apical segmental bronchus; RB10, posterior basal segmental bronchus of the right lower lobe; LB10, posterior basal segmental bronchus of the left lower lobe; AS, asthma; CVA, cough variant asthma; CTL, control group; M/F, Male/Female; WBC, white blood cell; EOS, eosinophil; EOS%, eosinophil percentage; WT, wall thickness; WT%, wall thickness percentage; WA, wall area; WA%, wall area percentage; c/, AS group vs. CTL group; §, CVA group vs. CTL group.

**TABLE 2:** Comparison of clinical data and CT parameters between the AS group and CVA group.

Variables	AS *n* = 51	CVA *n* = 50	t/χ^2^	*p*
Age	6.33 ± 2.37	6.22 ± 2.41	0.238	0.813
Sex (M/F)	33/18	28/22	0.008	0.371
BSA	0.81 ± 0.32	0.83 ± 0.26	−0.428	0.674
WBC	8.39 ± 3.19	8.59 ± 3.48	−0.299	0.766
EOS%	4.41 ± 5.37	3.05 ± 2.55	1.555	0.124
EOS	0.35 ± 0.51	0.24 ± 0.21	1.328	0.188
**Third-order bronchi of RB1**				
WA	12.76 ± 3.54	13.22 ± 3.40	−0.648	0.518
WT	1.03 ± 0.16	1.09 ± 0.15	−1.884	0.062
WT%	0.42 ± 0.07	0.44 ± 0.08	−0.086	0.931
WA%	0.66 ± 0.07	0.68 ± 0.09	−0.697	0.488
**Fourth-order bronchi of RB1**				
WA	8.49 ± 2.89	8.54 ± 2.24	0.896	0.372
WT	0.92 ± 0.19	0.94 ± 2.89	0.278	0.781
WT%	0.48 ± 0.08	0.49 ± 0.08	−0.193	0.847
WA%	0.73 ± 0.08	0.73 ± 0.09	−0.140	0.889
**Third-order bronchi of RB10**				
WA	13.22 ± 3.45	12.61 ± 3.25	−0.292	0.771
WT	1.02 ± 0.20	1.01 ± 0.20	0.217	0.828
WT%	0.40 ± 0.07	0.40 ± 0.07	−0.007	0.994
WA%	0.63 ± 0.09	0.63 ± 0.09	0.023	0.982
**Fourth-order bronchi of RB10**				
WA	9.61 ± 3.21	9.82 ± 3.33	−0.637	0.526
WT	0.95 ± 0.23	0.94 ± 0.20	−1.130	0.262
WT%	0.46 ± 0.09	0.45 ± 0.08	0.860	0.392
WA%	0.70 ± 0.09	0.69 ± 0.09	0.750	0.455
**Third-order bronchi of LB10**				
WA	13.66 ± 3.66	13.32 ± 3.51	−0.086	0.931
WT	1.01 ± 0.17	1.03 ± 0.18	−0.697	0.488
WT%	0.38 ± 0.07	0.40 ± 0.07	−1.206	0.231
WA%	0.62 ± 0.08	0.64 ± 0.09	−1.124	0.264
**Fourth-order bronchi of LB10**				
WA	9.60 ± 3.14	9.55 ± 2.59	0.896	0.372
WT	0.91 ± 0.21	0.96 ± 0.16	0.278	0.781
WT%	0.43 ± 0.09	0.45 ± 0.09	−0.698	0.487
WA%	0.67 ± 0.11	0.69 ± 0.11	−0.648	0.518
**Pulmonary function**				
FVC (%)	87.51 ± 16.84	94.57 ± 12.68	−1.522	0.136
FEV_1_ (%)	80.82 ± 21.94	93.06 ± 14.06	−2.165	0.036
FEV_1_/FVC (%)	91.12 ± 11.21	97.45 ± 6.44	−2.256	0.029
FEF_25_ (%)	68.60 ± 26.90	79.28 ± 18.70	−1.505	0.140
FEF_50_ (%)	57.28 ± 29.25	70.72 ± 19.97	−1.752	0.087
FEF_75_ (%)	47.38 ± 25.77	56.17 ± 18.84	−1.272	0.210
MMEF (%)	55.44 ± 28.62	67.06 ± 19.72	−1.544	0.130
Z_5_ (%)	133.50 ± 26.74	125.88 ± 36.60	0.837	0.406
R_5_ (%)	133.93 ± 26.56	136.62 ± 19.05	−0.426	0.672
R_20_ (%)	94.50 ± 22.14	98.48 ± 15.67	−0.758	0.452
R_5_-R_20_ (%)	32.86 ± 8.42	32.59 ± 12.51	0.090	0.929
Fres (Hz)	32.86 ± 8.42	32.59 ± 12.51	0.340	0.735
ΔX5 (kpa)	−0.08 ± 0.16	−0.02 ± 0.13	−1.434	0.158
FeNO	14.02 ± 11.12	14.40 ± 10.67	−0.145	0.885

RB1, right upper lobe apical segmental bronchus; RB10, posterior basal segmental bronchus of the right lower lobe; LB10, posterior basal segmental bronchus of the left lower lobe; AS, asthma; CVA, cough variant asthma; M/F, Male/Female; WBC, white blood cell; EOS, eosinophil; EOS%, eosinophil percentage; WT, wall thickness; WT%, wall thickness percentage; WA, wall area; WA%, wall area percentage; BSA, Body Surface Area; FVC, forced vital capacity; FEV_1_, forced expiratory volume in the first second; FEV_1_/FVC, forced expiratory volume in one second/forced vital capacity; FEF_25_, forced expiratory flow at 25% of FVC; FEF_50_, forced expiratory flow at 50% of FVC; FEF_75_, forced expiratory flow at 75% of FVC; MMEF, Maximal Mid-Expiratory Flow; Z_5_, respiratory impedance at 5 Hz; R_5_, respiratory resistance at 5 Hz; R_20_, respiratory resistance at 20 Hz; R_5_-R_20_, difference between R_5_ and R_20_; Fres, resonance frequency; X_5_, respiratory reactance at 5 Hz; FeNO, fractional exhaled nitric oxide.

Non-severe asthma group vs. control: the AS group had significantly higher values (*p* < 0.05) in EOS%, and WA, WT, WA%, WT% of the 3rd and 4th order bronchi of the RB1 and RB10, as well as WA and WT of the 3rd and 4th order bronchi of LB10. No significant differences were observed in age, sex, WBC, absolute EOS count, or in WA% and WT% of the 3rd and 4th order bronchi of LB10.

Cough-variant asthma group vs. control: the CVA group demonstrated significantly higher values (*p* < 0.05) in EOS%, and WT, WA%, WT% of the 3rd order bronchi of the RB1, as well as WA, WT, WA%, WT% of the 4th order bronchi of the RB1 and 3rd and 4th order of RB10 and LB10. No significant difference was found in age, sex, WBC, and absolute EOS count.

Non-severe asthma group vs. CVA group: a direct comparison between the AS and CVA groups revealed a significant difference in the lung function parameters FEV_1_ and FEV_1_/FVC (*p* < 0.05). However, no significant differences were observed in the other clinical characteristics or CT parameters listed above.

### 3.2 Comparison between children with and without small airway dysfunction (SAD)

Among the 101 patients in the combined AS and CVA groups, 94 (93.1%) successfully completed pulmonary function assessments and were included in the SAD and sub-analysis. Specifically, in the AS group (*n* = 45), 22 underwent spirometry and 23 underwent IOS; in the CVA group (*n* = 49), 21 underwent spirometry and 28 underwent IOS. Seven patients (6 in the AS group and 1 in the CVA group) were excluded from this sub-analysis due to their inability to perform the maneuvers. A comparison of clinical characteristics and CT parameters between SAD group and non-SAD group is presented in Table 3. The results demonstrated that the SAD group had significantly higher values for WA% in the RB1 subsegment, WT% in the RB1 subsegment, and WT% in the RB10 subsegment compared to the non-SAD group. No statistically significant differences were observed between the two groups for the remaining parameters.

**TABLE 3:** Comparison of clinical data and CT parameters between the SAD group and non-SAD group.

Variables	SAD *n* = 49	Non-SAD *n* = 45	t/χ^2^	*p*
Age	6.40 ± 2.23	6.28 ± 2.59	0.239	0.812
Sex (M/F)	30/19	26/19	0.116	0.734
BSA	0.87 ± 0.23	0.86 ± 0.22	0.357	0.722
WBC	8.52 ± 3.01	8.62 ± 3.78	−0.135	0.893
EOS%	4.25 ± 5.19	8.52 ± 3.01	1.031	0.305
EOS	8.62 ± 3.78	4.25 ± 5.19	1.319	0.191
FeNO	16.05 ± 11.69	12.87 ± 10.08	1.199	0.235
**Third-order bronchi of RB1**				
WA	13.26 ± 3.49	13.06 ± 3.53	0.272	0.786
WT	1.08 ± 0.16	1.05 ± 0.14	1.02	0.31
WT%	0.44 ± 0.07	0.43 ± 0.06	0.998	0.321
WA%	0.68 ± 0.08	0.67 ± 0.07	0.948	0.346
**Fourth-order bronchi of RB1**				
WA	8.93 ± 2.78	8.28 ± 2.46	1.009	0.316
WT	0.96 ± 0.18	0.89 ± 0.18	1.895	0.061
WT%	0.51 ± 0.07	0.47 ± 0.07	2.007	0.048
WA%	0.75 ± 0.07	0.72 ± 0.08	2.034	0.045
**Third-order bronchi of RB10**				
WA	12.91 ± 2.94	12.94 ± 3.51	0.095	0.925
WT	1.02 ± 0.16	0.99 ± 0.22	0.941	0.35
WT%	0.41 ± 0.07	0.38 ± 0.07	1.655	0.101
WA%	0.65 ± 0.08	0.61 ± 0.09	1.728	0.088
**Fourth-order bronchi of RB10**				
WA	9.55 ± 3.26	9.80 ± 3.29	−0.04	0.969
WT	0.96 ± 0.24	0.93 ± 0.18	1.067	0.289
WT%	0.47 ± 0.09	0.44 ± 0.07	2.014	0.047
WA%	0.72 ± 0.10	0.68 ± 0.08	1.86	0.067
**Third-order bronchi of LB10**				
WA	13.65 ± 3.58	13.32 ± 3.73	0.579	0.564
WT	1.05 ± 0.18	0.99 ± 0.18	1.62	0.109
WT%	0.41 ± 0.07	0.38 ± 0.07	1.803	0.075
WA%	0.64 ± 0.08	0.61 ± 0.09	1.797	0.076
**Fourth-order bronchi of LB10**				
WA	9.63 ± 2.99	9.25 ± 3.17	0.741	0.461
WT	0.95 ± 0.20	0.88 ± 0.22	1.507	0.136
WT%	0.46 ± 0.08	0.42 ± 0.10	1.643	0.104
WA%	0.70 ± 0.10	0.66 ± 0.12	1.664	0.1

SAD, small airway dysfunction; RB1, right upper lobe apical segmental bronchus; RB10, posterior basal segmental bronchus of the right lower lobe; LB10, posterior basal segmental bronchus of the left lower lobe; M/F, Male/Female; BSA, Body Surface Area; WBC, white blood cell; EOS, eosinophil; EOS%, eosinophil percentage; WT, wall thickness; WT%, wall thickness percentage; WA, wall area; WA%, wall area percentage.

### 3.3 Comparison between patients with and without obstructive ventilatory disorder (OVD)

Consistent with the SAD sub-analysis, the comparative analysis for OVD was conducted among the same cohort of 94 patients who successfully completed the pulmonary function test, and the 94 patients in the AS and CVA groups were categorized into OVD and non-OVD groups based on the presence or absence of an obstructive ventilatory disorder (obstructive ventilatory disorder) on spirometry or IOS. A comparative analysis of clinical data and CT parameters between the groups is summarized in Table 4. The OVD group demonstrated significantly elevated values for WT%, and WA% in the 4th order bronchi of RB10, as well as for WT, and WA% in the 4th order bronchi of LB10, compared to the non-OVD group. No statistically significant differences were found for the remaining parameters.

**TABLE 4:** Comparison of clinical data and CT parameters between the OVD group and non-OVD group.

Variables	OVD *n* = 59	Non-OVD *n* = 35	t/χ^2^	*p*
Age	6.16 ± 2.44	6.65 ± 2.33	−0.951	0.344
Sex (M/F)	32/27	24/11	1.874	0.171
BSA	0.89 ± 0.20	0.87 ± 0.22	0.351	0.517
WBC	8.94 ± 3.79	7.82 ± 2.33	1.577	0.118
EOS%	4.02 ± 5.03	3.46 ± 2.77	0.605	0.547
EOS	0.34 ± 0.48	0.24 ± 0.18	1.151	0.253
FeNO	14.46 ± 10.78	14.76 ± 11.60	−0.110	0.912
**Third-order bronchi of RB1**				
WA	13.17 ± 3.57	13.16 ± 3.43	0.005	0.996
WT	1.08 ± 0.15	1.08 ± 0.15	0.966	0.336
WT%	0.44 ± 0.07	0.42 ± 0.07	1.286	0.202
WA%	0.68 ± 0.07	0.66 ± 0.08	1.351	0.18
**Fourth-order bronchi of RB1**				
WA	8.72 ± 2.67	8.57 ± 2.42	0.256	0.798
WT	0.95 ± 0.18	0.92 ± 0.16	1.071	0.287
WT%	0.50 ± 0.07	0.47 ± 0.08	1.586	0.116
WA%	0.74 ± 0.07	0.72 ± 0.08	1.621	0.108
**Third-order bronchi of RB10**				
WA	12.91 ± 3.45	13.40 ± 3.13	−0.67	0.505
WT	1.03 ± 0.20	1.00 ± 0.18	0.845	0.401
WT%	0.41 ± 0.07	0.38 ± 0.06	2.200	0.03
WA%	0.65 ± 0.09	0.61 ± 0.08	0.655	0.036
**Fourth-order bronchi of RB10**				
WA	9.87 ± 3.38	9.63 ± 3.23	1.261	0.75
WT	0.99 ± 0.22	0.89 ± 0.21	1.160	0.059
WT%	0.48 ± 0.08	0.42 ± 0.08	3.294	0.001
WA%	0.72 ± 0.08	0.66 ± 0.09	3.268	0.002
**Third-order bronchi of LB10**				
WA	13.63 ± 3.67	13.52 ± 3.57	13.638	0.890
WT	1.04 ± 0.17	1.00 ± 0.18	13.527	0.318
WT%	0.41 ± 0.06	0.38 ± 0.08	1.045	0.164
WA%	0.64 ± 0.08	0.61 ± 0.10	1.005	0.148
**Fourth-order bronchi of LB10**				
WA	10.08 ± 2.97	9.14 ± 2.82	0.616	0.172
WT	0.98 ± 0.18	0.88 ± 0.19	16.173	0.028
WT%	0.46 ± 0.07	0.42 ± 0.09	15.667	0.056
WA%	0.71 ± 0.08	0.66 ± 0.11	1.259	0.046

OVD, obstructive ventilatory disorder; RB1, right upper lobe apical segmental bronchus; RB10, posterior basal segmental bronchus of the right lower lobe; LB10, posterior basal segmental bronchus of the left lower lobe; M/F, Male/Female; BSA, Body Surface Area; WBC, white blood cell; EOS, eosinophil; EOS%, eosinophil percentage; WT, wall thickness; WT%, wall thickness percentage; WA, wall area; WA%, wall area percentage.

### 3.4 Correlation analysis between CT parameters and pulmonary function indices

The CT parameters (WA, WT, WA%, and WT%) from the 3rd and 4th generation bronchi of RB1, RB10, and LB10 were averaged. Multiple linear regression analyses were then performed to assess the associations between these CT parameters and the pulmonary function indices. The results, presented in Supplementary Tables 1, 2, revealed no significant associations between the CT parameters and pulmonary function measures.

## 4 Discussion

Previous studies have found that early, functional remodeling indicators (such as airway wall thickness, inflammatory factor expression) mediated by eosinophilic inflammation have potential for improvement, but structural changes that have already formed (such as stromal remodeling, long-term collagen deposition) may be difficult to reverse (12). Long-term inhaled glucocorticoid treatment can reduce the thickness of the reticular basement membrane and inhibit eosinophilic inflammation in patients with asthma, which may retard the progression of airway remodeling (13). Hence, early detection and standardized treatment are crucial for preventing the progression of airway remodeling in patients with asthma and improving the reversibility of remodeling indicators. However, traditional methods for assessing airway remodeling in asthma patients involve pathological biopsy, which is difficult, invasive, and impractical in clinical practice. This study systematically compared CT imaging parameters, pulmonary function, and EOS counts among children with AS, CVA, and a healthy control group. The results revealed that the WA, WT, and their proportions like WA% and WT% at specific bronchial segments such as RB1, RB10, and LB10 were significantly higher in both the AS and CVA groups compared to the healthy control (*p* < 0.05). Crucially, our study demonstrated an increase in the absolute measurements of WA and WT themselves in the AS and CVA groups, not merely in the relative percentages (WA% and WT%). This indicated that the airway wall is indeed thickened, rather than merely an indirect result of bronchospasm leading to a reduction in the lumen diameter. These findings suggest that airway remodeling may commence in the early stages of childhood asthma, and is not exclusive to severe disease. Previous studies on severe and refractory asthma have established that HRCT parameters indicative of bronchial wall thickening, such as WT%, can serve as characteristic markers of severe disease (7). Our study, however, observed this bronchial wall thickening phenomenon even in non-severe pediatric cases. This suggests that the process of airway remodeling may initiate at lower severity levels across the disease spectrum. Although validation via biopsy was not performed, our findings indicate that these HRCT parameters hold potential for preliminary assessment and dynamic monitoring of this condition.

Notably, the AS group demonstrated more severe lung function impairment, including lower FEV1 and FEV1/FVC ratios, than the CVA group. This indicated a greater degree of airway obstruction, which aligns with previous studies (14, 15). However, there were no significant differences in the CT parameters between the CVA and AS groups, and the values in both groups were significantly higher than those in the CTL group. These findings suggest that despite differences in clinical presentation and lung function, these two asthma subtypes share similarities in structural airway remodeling as assessed by CT. This provides indirect support for the view that airway hyperresponsiveness is the primary mechanism in CVA, but that airway remodeling also contributes to its pathological process (16).

This study found that although CT-derived airway parameters were elevated in patient groups, they did not demonstrate a significant correlation with pulmonary function measures. This apparent lack of association should be interpreted with caution, as the retrospective design, relatively small cohort size, and restriction of imaging analysis to selected segmental airways using limited morphometric indices (WT and WA) may reduce the ability to detect structure–function relationships. Importantly, prior work supports a physiological link between airway structure and clinical outcomes. Small airways are recognized to play a critical role in early disease pathophysiology and may disproportionately influence lung mechanics (17), while remodeling of central airways has also been shown to contribute to measurable functional impairment (18, 19). Therefore, the absence of a significant correlation in the present study likely reflects methodological and sampling constraints rather than a true dissociation between airway structure and lung function.

Compared to the non-OVD group, children in the OVD group exhibited significantly higher values of WA% in the 4th order bronchi of RB10, as well as WT and WA% in the 4th order bronchi of LB10. No statistically significant differences were observed in the remaining parameters between the two groups. This selective difference may be attributed to our study cohort, which primarily consisted of non-severe asthmatic children. Since CT measurements were taken at generations 3–4 airways, and asthmatic involvement in early stages tends to affect more distal airways, the significant wall thickening in the OVD group at generation 4 aligns with this pathological pattern. Furthermore, the overall baseline lung function in our non-severe cohort was relatively preserved (e.g., mean FEV1 > 80% predicted in both AS and CVA groups). This suggests that the structural changes may not yet be severe or extensive enough to broadly impact airway function. Consequently, longer-term longitudinal follow-up is required to further elucidate the relationship between these CT structural parameters and functional measures.

Prior studies have indicated that elevated peripheral blood EOS levels are associated with poorer lung function outcomes. Patients with eosinophilic inflammation face a higher risk of lung function decline and more severe airway remodeling over time (20). In contrast, our study found that while the EOS% was higher in the AS and CVA groups than in the control group, the absolute EOS count was not significantly elevated. We speculate that this discrepancy may be attributed to our cohort consisting solely of non-severe or non-refractory asthma cases. Furthermore, standardized reference ranges for EOS counts in the pediatric population are currently lacking.

This study still has limitations. First, although airway measurements were standardized to predefined segmental bronchi (RB1, RB10, and LB10) to enhance measurement reliability, this sampling strategy represents proxy measurements rather than a comprehensive assessment of airway remodeling throughout the entire bronchial tree. Airway structural changes in asthma may exhibit regional heterogeneity, particularly in more distal or smaller airways that were not evaluated in this study. Second, its cross-sectional design limits the ability to infer its causality. Future work will involve follow-up of this cohort to track the progression of airway remodeling following early, standardized management and treatment. Additionally, longitudinal studies with repeated CT scans are planned to delineate these dynamic changes.

## 5 Conclusion

In summary, this study provides imaging evidence of airway remodeling in children with non-severe asthma, highlighting the value of CT in its early detection. By innovatively including data from CVA patients, we revealed that despite differences in clinical presentation and pulmonary function, CVA shares a similar phenotype of airway wall thickening with classic asthma. Although no significant correlation was established between CT parameters and lung function, this may reflect a characteristic of the early disease stage. Further exploration of the role of thoracic imaging in the dynamic monitoring of asthma patients holds promise for optimizing clinical management strategies. Consequently, our subsequent work will involve longitudinal follow-up to assess the ability of early detection and intervention to mitigate or prevent the progression of airway remodeling.

## Data Availability

The original contributions presented in the study are included in the article/Supplementary material, further inquiries can be directed to the corresponding author.
