# Supplementary material for: CT imaging in children with non-severe asthma and cough-variant asthma: functional spasm or structural remodeling?
Source: Front Med (Lausanne). 2026 Mar 16;13:1755012. doi: 10.3389/fmed.2026.1755012 (PMC13033675; doi:10.3389/fmed.2026.1755012)
Supplement: Supplementary file 1 [file Table_1.DOCX]

Table 1 Correlation of Third-Order Bronchial CT Characteristics with Pulmonary Function

|  | Third-order bronchi | | | | | | | | | | | |
| --- | --- | --- | --- | --- | --- | --- | --- | --- | --- | --- | --- | --- |
|  | WA | | | WT | | | WT% | | | WA% | | |
|  | standardized β coefficients | t | p | standardized β coefficients | t | p | standardized β coefficients | t | p | standardized β coefficients | t | p |
| FVC | 2.358 | 1.750 | 0.114 | -0.201 | -0.141 | 0.891 | -3.104 | -1.872 | 0.094 | -2.997 | -1.818 | 0.102 |
| FEV_1_ | -4.539 | -2.223 | 0.053 | -0.710 | -0.330 | 0.749 | 4.346 | 1.730 | 0.118 | 4.234 | 1.695 | 0.124 |
| FEV_1_/FVC | 1.108 | 1.408 | 0.193 | -0.314 | -0.379 | 0.714 | -1.826 | -1.885 | 0.092 | -1.757 | -1.824 | 0.101 |
| FEF_25_ | -0.584 | -1.257 | 0.241 | 0.229 | 0.467 | 0.651 | 1.002 | 1.754 | 0.113 | 0.925 | 1.627 | 0.138 |
| FEF_50_ | -1.662 | -1.084 | 0.307 | -1.073 | -0.663 | 0.524 | 0.612 | 0.324 | 0.753 | 0.496 | 0.265 | 0.797 |
| FEF_75_ | 1.234 | 1.472 | 0.175 | 2.362 | 2.671 | 0.026 | 2.165 | 2.099 | 0.065 | 2.216 | 2.161 | 0.059 |
| MMEF | 2.390 | 1.073 | 0.311 | -0.593 | -0.252 | 0.806 | -4.015 | -1.465 | 0.177 | -3.908 | -1.434 | 0.185 |
| Z_5_ | -0.543 | -0.422 | 0.677 | -1.886 | -1.806 | 0.085 | -1.531 | -1.260 | 0.221 | -1.612 | -1.317 | 0.201 |
| R_5_ | 0.646 | 0.526 | 0.604 | 2.195 | 2.197 | 0.039 | 1.864 | 1.604 | 0.123 | 1.946 | 1.663 | 0.110 |
| R_20_ | 0.376 | 1.023 | 0.317 | -0.009 | -0.030 | 0.976 | -0.366 | -1.054 | 0.303 | -0.327 | -0.936 | 0.359 |
| R_5_-R_20_ | 0.342 | 1.066 | 0.298 | -0.073 | -0.282 | 0.781 | -0.456 | -1.505 | 0.147 | -0.418 | -1.372 | 0.184 |
| Fres | -0.008 | -0.028 | 0.978 | 0.335 | 1.398 | 0.176 | 0.398 | 1.428 | 0.167 | 0.369 | 1.313 | 0.203 |
| ΔX_5_kpa | 0.177 | 0.563 | 0.579 | -0.364 | -1.427 | 0.168 | -0.657 | -2.216 | 0.037 | -0.619 | -2.072 | 0.050 |

Note: WT, wall thickness; WT%, wall thickness percentage; WA, wall area; WA%, wall area percentage; FVC, Forced Vital Capacity; FEV_1_, Forced Expiratory Volume in the first second; FEV_1_/FVC, Forced Expiratory Volume in One Second/Forced Vital Capacity; FEF_25_, Forced Expiratory Flow at 25% of FVC; FEF_50_, Forced Expiratory Flow at 50% of FVC; FEF_75_, Forced Expiratory Flow at 75% of FVC; MMEF, Maximal Mid-Expiratory Flow; Z_5_, Respiratory Impedance at 5 Hz; R_5_, Respiratory Resistance at 5 Hz; R_20_, Respiratory Resistance at 20 Hz; R5-R20, Difference between R_5_ and R_20_; Fres, Resonance Frequency; X_5_, Respiratory Reactance at 5 Hz.

Table 2 Correlation of Fourth-order Bronchial CT Characteristics with Pulmonary Function

|  | Fourth-order bronchi | | | | | | | | | | | |
| --- | --- | --- | --- | --- | --- | --- | --- | --- | --- | --- | --- | --- |
|  | WA | | | WT | | | WT% | | | WA% | | |
|  | standardized β coefficients | t | p | standardized β coefficients | t | p | standardized β coefficients | t | p | standardized β coefficients | t | p |
| FVC | 2.043 | 0.972 | 0.356 | 0.676 | 0.309 | 0.764 | -1.727 | -1.219 | 0.254 | -1.727 | -1.219 | 0.254 |
| FEV1 | -2.996 | -0.941 | 0.371 | -1.279 | -0.386 | 0.709 | 2.040 | 0.950 | 0.367 | 2.040 | 0.950 | 0.367 |
| FEV1/FVC | 0.287 | 0.234 | 0.820 | -0.976 | -0.764 | 0.465 | -2.064 | -2.492 | 0.034 | -2.064 | -2.492 | 0.034 |
| FEF_25_ | 0.319 | 0.441 | 0.670 | 1.651 | 2.190 | 0.056 | 2.175 | 4.452 | 0.002 | 2.175 | 4.452 | 0.002 |
| FEF_50_ | -0.729 | -0.305 | 0.767 | 0.103 | 0.041 | 0.968 | 1.267 | 0.786 | 0.452 | 1.267 | 0.786 | 0.452 |
| FEF_75_ | -0.007 | -0.005 | 0.996 | 0.553 | 0.406 | 0.694 | 0.709 | 0.804 | 0.442 | 0.709 | 0.804 | 0.442 |
| MMEF | 1.296 | 0.373 | 0.718 | -0.869 | -0.240 | 0.816 | -2.986 | -1.274 | 0.235 | -2.986 | -1.274 | 0.235 |
| Z_5_ | -1.987 | -1.520 | 0.144 | -2.134 | -1.768 | 0.092 | -1.308 | -1.033 | 0.313 | -1.308 | -1.033 | 0.313 |
| R_5_ | 2.218 | 1.767 | 0.092 | 2.450 | 2.113 | 0.047 | 1.659 | 1.364 | 0.187 | 1.659 | 1.364 | 0.187 |
| R_20_ | -0.198 | -0.533 | 0.599 | -0.225 | -0.658 | 0.518 | -0.255 | -0.709 | 0.486 | -0.255 | -0.709 | 0.486 |
| R_5_-R_20_ | -0.042 | -0.130 | 0.898 | -0.080 | -0.268 | 0.792 | -0.221 | -0.705 | 0.488 | -0.221 | -0.705 | 0.488 |
| Fres | 0.325 | 1.092 | 0.287 | 0.391 | 1.423 | 0.169 | 0.321 | 1.116 | 0.277 | 0.321 | 1.116 | 0.277 |
| ΔX5kpa | -0.051 | -0.163 | 0.872 | -0.271 | -0.934 | 0.361 | -0.487 | -1.602 | 0.124 | -0.487 | -1.602 | 0.124 |

Note: WT, wall thickness; WT%, wall thickness percentage; WA, wall area; WA%, wall area percentage; FVC, Forced Vital Capacity; FEV_1_, Forced Expiratory Volume in the first second; FEV_1_/FVC, Forced Expiratory Volume in One Second/Forced Vital Capacity; FEF_25_, Forced Expiratory Flow at 25% of FVC; FEF_50_, Forced Expiratory Flow at 50% of FVC; FEF_75_, Forced Expiratory Flow at 75% of FVC; MMEF, Maximal Mid-Expiratory Flow; Z_5_, Respiratory Impedance at 5 Hz; R_5_, Respiratory Resistance at 5 Hz; R_20_, Respiratory Resistance at 20 Hz; R5-R20, Difference between R_5_ and R_20_; Fres, Resonance Frequency; X_5_, Respiratory Reactance at 5 Hz.
